# Antiviral Perspectives for Chikungunya Virus

**DOI:** 10.1155/2014/631642

**Published:** 2014-05-15

**Authors:** Deepti Parashar, Sarah Cherian

**Affiliations:** National Institute of Virology, 20-A Dr. Ambedkar Road, Pune 411001, India

## Abstract

Chikungunya virus (CHIKV) is a mosquito-borne pathogen that has a major health impact in humans and causes acute febrile illness in humans accompanied by joint pains and, in many cases, persistent arthralgia lasting for weeks to years. CHIKV reemerged in 2005-2006 in several parts of the Indian Ocean islands and India after a gap of 32 years, causing millions of cases. The re-emergence of CHIKV has also resulted in numerous outbreaks in several countries in the eastern hemisphere, with a threat to further expand in the near future. However, there is no vaccine against CHIKV infection licensed for human use, and therapy for CHIKV infection is still mainly limited to supportive care as antiviral agents are yet in different stages of testing or development. In this review we explore the different perspectives for chikungunya treatment and the effectiveness of these treatment regimens and discuss the scope for future directions.

## 1. Introduction 

The reemergence of chikungunya virus (CHIKV) in many parts of the world is a significant public health concern. Since the 2005-2006 chikungunya fever epidemic in the Indian Ocean island of La Réunion [1, 2] millions of people in more than 40 countries including India, Malaysia, Indonesia, Thailand, Singapore, the United States, and some European countries have been infected [3–5].

The CHIKV was first isolated from febrile individuals in Tanzania in 1952 [6, 7]. Initially, researchers classified it among viruses such as Sindbis and Semliki Forest virus. Later studies characterized it as an alpha virus that belongs to the family Togaviridae. CHIKV is an enveloped virus with a genome approximately 11.8 kb in size. It consists of a single stranded, positive sense RNA genome with two open reading frames (ORFs) [8]. The one at the 5′ end encodes four nonstructural proteins (nsP1–nsP4) and the second ORF at the 3′ end encodes the structural proteins, the capsid (C), envelope glycoproteins E1 and E2, and two small cleavage products (E3, 6K).

CHIKV is transmitted to humans by several species of mosquitoes, with* Aedes aegypti* and* A. albopictus *being the two main vectors. The symptoms generally start 4–7 days after the bite. Acute infection lasts for 1–10 days and is characterized by abrupt onset of fever, headache, fatigue, nausea, vomiting, rash, myalgia, and severe arthralgia. Painful polyarthralgia is the typical symptom and may persist in ~10% of cases for several months causing serious economic and social impacts on both the individual and the affected communities in some instances [9].

Thus far, due to the lack of licensed vaccines or effective antivirals against CHIKV, most of the treatment regimens are symptomatic and based on the clinical manifestations. Nonsalicylate analgesics and nonsteroidal anti-inflammatory drugs are most commonly used for symptomatic relief [10]. Immune-based control strategies have also been tested and have shown effectiveness. The elaboration of mouse [11, 12] and nonhuman primate models [13, 14] together with antivirals currently undergoing clinical trials and new approaches involving screening of libraries of small molecules and synthetic compounds with antiviral potential as well as natural products of plants [15] is further providing the stimulus for improving the development of antiviral candidates.

Recently Rashad et al. [16] have presented a detailed review of the current medicinal chemistry aspects supporting the development of chemotherapeutics targeting the CHIKV through drug discovery and design. On the other hand, this review is an attempt to compile and explore the various strategies that have been tried for the management and treatment of CHIKV from a virological perspective and further discuss possible directions for the future.

## 2. Chemical Compounds

We describe here some of the commonly used nonsteroidal anti-inflammatory drugs (NSAIDs) and nonsalicylate analgesics that have played a major role in symptomatic treatment of the chikungunya disease along with some newer compounds that have been recently tested against the CHIKV infection. 

*(A) Symptomatic Treatment Strategies*

*Nonsteroidal Anti-Inflammatory Drugs.* Current treatments for viral arthropathies rely mainly on NSAIDs though these often provide only partial relief [17]. A number of patients who experienced chronic rheumatic symptoms following infection with CHIKV during the 2005-2006 La Réunion outbreak were successfully treated with methotrexate (MTX) [18]. MTX, which was originally developed as a chemotherapeutic, forms the basis of most rheumatoid arthritis (RA) treatment regimens due to its anti-inflammatory effects at low doses [19]. Despite this, details of its anti-inflammatory mechanism and its effect on acute viral induced arthritis remain unclear. Some rare chronic patients did benefit from MTX but seem to be in a different diagnostic class “post-chikungunya rheumatism” [20]. However, treatment efficacy is hard to evaluate when multiple diseases are present. In a study of post-CHIKV chronic arthritis in Maharashtra, a south-western state of India, antirheumatic drugs including Sulfasalazine and MTX were needed for effective treatment [21]. In a recent study aimed at understanding the anti-inflammatory mechanism of MTX and its effect on acute viral induced arthritis, a mouse model of Ross River virus-induced inflammatory disease demonstrated that MTX treatment caused early onset of disease through increased monocyte production [22]. The findings of MTX effectiveness thus seem to be contradictive.

Further, although adverse effects of NSAIDs occur in only a small proportion of users, the widespread use of these drugs has resulted in serious gastrointestinal complications in a substantial overall number of affected persons. Selective COX-II inhibitors such as rofecoxib, celecoxib, and parecoxib have shown consistently comparable efficacy to that of conventional NSAIDs in patients with rheumatoid arthritis, with a significantly reduced propensity to cause gastrointestinal toxicity. The safety benefits of COX-II inhibitors given alone appear similar to combined therapy with conventional NSAIDs and gastroprotective agents, justifying the consideration of such inhibitors as first-line therapy in patients requiring long-term pain control [23].

*Nonsalicylate Analgesics* (*Paracetamol, Acetaminophen, Morphine, and Traditional Herbal Medicine).* Most data available regarding the use of nonsalicylate analgesics against the CHIKV is from the follow-up of La Réunion patients. Based on clinical manifestations, the analgesic drug Paracetamol was the most used (in 95.4% of treatments) and often was combined with NSAIDs. The wide use of paracetamol led to the emergence of severe liver afflictions diagnosed during the epidemic, particularly when doses >3 g/day were taken [24]. The hepatotoxicity of paracetamol in combination with interferon and vinblastine has also been reported [25]. Further, studies have shown that acetaminophen (APAP), another analgesic which is commonly used for the relief of fever, and flu-like symptoms, modulates the transcriptional response to recombinant interferon-beta [26]. This also suggests that the use of this class of drugs might need some caution. Morphine was seldom used in the La Réunion although it was reported to be effective during early treatments in a period when the CHIKV was first discovered [27]. Plants are an integral part of the Réunion Island pharmacopeia and therefore were mainly used during the outbreaks to treat fever, pain, and inflammation. Some plant species such as* Fernelia* spp. are also known for their antiviral properties. However, the efficacy of these plants or association of plants has not been studied, particularly for the hepatotoxic risk that might be present when taken with paracetamol [24].

*Steroids.* Corticoids were prescribed to treat arthralgia (27.7% of cases), particularly invalidating forms during the chikungunya disease in the Reunion Island hospital staff [24]. A study carried out in South India during the chikungunya epidemic in 2007 with the objective of evolving a uniform treatment protocol demonstrated that addition of prednisolone to aceclofenac reduced pain and improved the quality of life in patients with acute chikungunya arthritis, compared to aceclofenac given alone in the management of early chikungunya fever. Their studies thus recommended coadministration of low-dose systemic corticosteroids with NSAIDs as the best regimen in treating acute chikungunya cases with arthralgia [28]. In another report, regarding a case of post chikungunya reversible demyelinating encephalitis that was presented with vertigo, dysarthria, and ataxia, there was complete clinical as well as radiological improvement with steroids [29]. However, corticosteroid use during acute viral arthritis is considered to be contraindicated as a result of the risk of immunosuppression causing enhanced infection and disease exacerbation [30].

*(B) Specific Chemical Compounds Tested against CHIKV*

### 2.1. Chloroquine

Chloroquine was first reported to inhibit Sindbis virus (SINV) and Semliki Forest Virus (SFV) infectivity* in vitro *more than 35 years ago [31–35], but studies in mice suggested that the drug might enhance viral replication and aggravate the disease [36]. However, chloroquine was found to be effective in treating chronic CHIKV-induced arthralgia with the possible mechanism attributed most likely to its anti-inflammatory properties that are adapted for the treatment of some autoimmune illnesses [37]. Recent research on the efficacy of chloroquine has focused on the dosage used to treat acute CHIKV infections [38, 39]. To advance the studies with chloroquine and CHIKV, a double blind placebo-controlled randomized trial [38, 40] was conducted in the Réunion Island. No statistical difference was observed between the chloroquine and placebo groups, either in mean duration of febrile arthralgia or rate of decrease of viraemia. However, the number of patients included in the study was too small to draw definitive conclusions regarding the efficacy of the chloroquine treatment. In another* in vitro* study [41] since chloroquine exerted its antiviral effects in all the three modes of treatment (pretreatment, concurrent, and posttreatment), it was suggested that it has prophylactic and therapeutic potentials. However, in a recent controlled evaluation of chloroquine in treating patients with early persistent musculoskeletal pain and arthritis following CHIKV infection, selected cytokine assays that were carried out to study the inflammatory nature and the response of the symptoms to it, do not support a meaningful therapeutic role of oral chloroquine in the management of the symptoms studied [42]. Further, the studies carried out by Padmakumar et al. [28] also suggested that coadministration of hydroxychloroquine with NSAIDs in acute stages of the disease does not offer any additional benefits.

### 2.2. Ribavirin and 6-Azauridine

Ribavirin has shown wide* in vitro* inhibitory activity against RNA viruses with different modes of action depending on the virus [40]. Some of the suggested mechanisms of action are through the inhibition of IMP dehydrogenase, error catastrophe mechanism, by causing mutations and by interaction with viral polymerase [43]. Ravichandran and Manian [44] determined whether antiviral treatment would make any difference in those patients who continue to have arthritis even after two weeks after the febrile episode. Preliminary observations showed that ribavirin may have a direct antiviral activity against chikungunya. Compared to ribavirin, 6-azauridine, a broad-spectrum antimetabolite, was more effective against CHIKV and showed a similar antiviral activity against SFV [45, 46]. Further, interferon-alpha and ribavirin in combination also showed a synergistic effect on the* in vitro *inhibition of CHIKV [46]. Human infection with CHIKV appears to induce immunological dysfunction. Though interferon usually boosts the immune response, caution in its usage is recommended until more extensive nonhuman primate models have been studied. Polyethylene glycol-conjugated (pegylated, PEG) alpha interferon appears to be an effective treatment against infection with Venezuelan equine encephalitis virus (VEEV) and has profound effects on the host immune response to infection [47]. Treatment of VEEV-infected BALB/c mice with PEG IFN-*α* resulted in a greatly enhanced survival from either a subcutaneous or an aerosol infection. Treatment, results in a number of changes to the immune response characteristics normally associated with VEEV infection including increased macrophage activation, absence of splenic CD4, CD8, and B cells by day 2 post infection normally associated with VEEV infection, and reduction in the high tumor necrosis factor alpha production by macrophages.

In an animal model study for CHIKV, Couderc et al. [11] have demonstrated that adult mice with a partially or totally abrogated type-I IFN pathway develop a mild or severe infection; thus, it might be justified to test pegylated alpha interferon on CHIKV and other pathogenic alphaviruses.

### 2.3. Arbidol

The antiviral drug arbidol (ARB) was originally developed at the Russian Research Chemical and Pharmaceutical Institute about 20 years ago. This drug has been used in Russia for prophylaxis and treatment of acute respiratory infection including influenza from 1990. It has been shown that ARB exhibits a wide range of activities against a number of RNA and DNA enveloped/nonenveloped viruses.* In cellulo,* antiviral ARB activity against CHIKV has been investigated [48]. This compound was found to present potent inhibitory activity against the virus propagated onto immortalized primary human fibroblast and vero cells. Their study also demonstrated the growth of an ARB resistant CHIKV mutant possessing a single amino acid substitution (G407R) localized in the E2 envelope protein. Though this study helps explain the mechanism of action of arbidol, it also brings out the point that ARB usage may be impaired as* in vivo *there could be selection of a resistant virus that is able to be disseminated.

### 2.4. Harringtonine

In a recent study, harringtonine, a cephalotaxine alkaloid, displayed potent inhibition of CHIKV infection with minimal cytotoxicity. Treatment of harringtonine against SINV, a related alphavirus, suggested that harringtonine could inhibit other alphaviruses as well. [49]. It was indicated that harringtonine inhibited an early stage of the CHIKV replication cycle which occurred after viral entry into cells. Interestingly, harringtonine displayed greater potency with a CHIKV strain carrying the E1 A226V mutation as compared with a strain carrying the wild type E1.

Table 1 presents a summary of the assays used, the hypothesized target, and the pros and cons of the compounds described above.

## 3. Inhibitors Based on RNA-Mediated Interference

RNA interference (RNAi) is a posttranscriptional process triggered by the introduction of double-stranded RNA (dsRNA) which leads to gene silencing in a sequence-specific manner. The method is based on targeting specific viral proteins which consequently leads to the shutdown of the protein expression process, thereby stopping viral replication. RNAi mediated inhibition of viral replication has emerged as a promising antiviral strategy. The small interfering RNA (siRNA) and small hairpin RNA (shRNA) molecules are central to RNA interference.

Small interfering RNAs (siRNAs) are formed by cleaving the dsRNAs by the RNase III family member, Dicer, into 19–23 nucleotide (nt) fragments with 5′ phosphorylated ends and 2-nt unpaired and unphosphorylated 3′ ends. The processing of the target RNA is achieved by endonuclease argonaute 2 (Ago 2) which is further incorporated into the RNAi specificity complex (RISC). On the other hand, plasmid-expressed short hairpin RNA (shRNA) requires the activity of endogenous exportin 5 and Ago2 (Argonaute 2) that forms a dimer with Dicer, which then receives the shRNA. The shRNA is cleaved in one step by Dicer generating a 19–23 nt duplex siRNA with 2 nt 3′ overhangs. The siRNAs guide RISC to the target mRNA. RISC delivers the mRNA to cytoplasmic foci named processing bodies (P-bodies or GW-bodies), wherein mRNA decay factors are concentrated. The target mRNA is cleaved by Ago2 and degraded.

### 3.1. Small Interfering RNA (siRNA) Molecules

The therapeutic application of siRNA for the inhibition of CHIKV replication has been examined in vero cells [51]. siRNAs against the conserved regions of nsP3 and E1 genes of CHIKV have been designed and siRNA activity was assessed by detecting both the infectious virus and its genome. The titers of the virus yield in the supernatant were determined at 24 and 48 hours postinfection. Results showed reduction of virus titre by ~1.5 log10- ~2.5 log10 in comparison to the control. The study thus showed limited success and further needs the investigation of these siRNA sequences in an* in vivo *model for their ultimate therapeutic application. Studies have also been conducted at National Institute of Virology, Pune, India, by designing eight siRNAs, either targeting the E2 or the ns1 genes of CHIKV. The efficiency of these siRNAs to inhibit the CHIKV production was assessed in vero cells. Two of the siRNAs were able to reduce CHIKV E3 transcripts by ~5log10 and ~2.5log10 when cells were transfected after 1 hour post infection. 100 pmol of siRNA was found optimum in perturbing CHIKV production. The combination of these siRNAs was found to be very effective in inhibiting the production of CHIKV. Importantly, these siRNAs could inhibit the CHIKV replication in Swiss albino mice when administered 72 hours after viral infection. A siRNA dose of 1 mg/kg body weight (1700 pmol) was found optimal to inhibit the CHIKV replication in Swiss albino mice. Our findings suggested that RNAi is capable of silencing sequence-specific genes of CHIKV and might constitute a new therapeutic strategy for CHIKV infection [52].

### 3.2. Small Hairpin RNA (shRNA) Molecules

The efficacy of plasmid-based short hairpin RNA (shRNA) against CHIKV replication in three CHIKV-permissive cell lines, namely, HeLa, RD, and BHK cells has been investigated. Cell clones expressing shRNAs against CHIKV E1 and nsP1 genes displayed significant inhibition of infectious CHIKV production, while shRNA capsid demonstrated a modest inhibitory effect as compared to scrambled shRNA cell clones and nontransfected cell controls. Analysis of CHIKV E2 protein expression and visualization of shRNA (E1 and nsP1) cell clones collectively demonstrated similar inhibitory trends against CHIKV replication. shRNA E1 showed non cell-type specific anti-CHIKV effects and broad-spectrum silencing against different geographical strains of CHIKV. Furthermore, shRNA E1 clones did not exert any inhibition against dengue virus and SINV replication, thus indicating the high specificity of shRNA against CHIKV replication. Moreover, no shRNA-resistant CHIKV mutant was generated after 50 passages of CHIKV in stable cell clones. More importantly, strong and sustained anti-CHIKV protection was conferred in suckling mice pretreated with different concentrations (3.1, 9.5 and 19 pmol) of shRNA (E1). Results have thus suggested the promising efficacy of anti-CHIKV shRNAs, in particular, plasmid-shRNA E1, as a novel antiviral strategy against CHIKV infection [53].

## 4. Cellular Factors

Another approach to inhibiting the CHIKV infection is by targeting cellular factors such as proteases that are involved in the processing of the CHIKV particles and cellular receptors that may have a role in viral replication, or in another way by the induction of immune-based cellular enzymes that possess antiviral activity.

### 4.1. Furin Inhibitors

Molecules that inhibit alphavirus entry into susceptible cells by blocking the intracellular cleavage of viral envelope glycoproteins have been investigated. CHIKV infection of cultured human cells was shown to be inhibited by impairing the maturation of the CHIKV E2 surface glycoprotein using the furin inhibitor peptide, decanoyl-RVKR-chloromethyl ketone [54]. Another study reported the possible inhibition of the replication of VEEV using polyclonal antibodies to laminin-binding protein [55].

### 4.2. 2′,5′-Oligoadenylate Synthetase (OAS3)

CHIKV is highly sensitive to the antiviral activity of Type-I interferons (IFN-*α*/*β*). Bréhin et al. [56] investigated the role of IFN-induced 2′,5′-oligoadenylate synthetase (OAS) family in innate immunity to CHIKV. They have established inducible human epithelial HeLa cell lines expressing either the large form of human OAS and OAS3 or the genetic variant OAS3-R844X which is predicted to lack about 20% of the OAS3 protein from the carboxy terminus. HeLa cells respond to ectopic OAS3 expression by efficiently inhibiting CHIKV growth. The characteristic of the antiviral effect was a blockade in early stages of virus replication. Thus, OAS3 pathway may represent a novel antialphaviral mechanism by which IFN-*α*/*β* controls CHIKV growth. HeLa cells expressing the truncated form of OAS3 were less resistant to CHIKV infection, raising the question on the involvement of OAS3 genetic polymorphism in human susceptibility to alphavirus infection [56]. In another study [57] a CHIKV variant exhibiting remarkable resistance towards the antiviral activity of OAS3, through an enhancement of viral RNA replication, was identified. It was shown that a single amino acid change in the E2 glycoprotein allows the rescue of viral growth in OAS3 expressing HeLa cells by acting on the early stages of viral life cycle. These observations also provide a new insight into the role of E2 into the pathogenicity of CHIKV in human cells [57].

### 4.3. Cellular IMPDH Enzyme

Mycophenolic acid (MPA) is a weak organic acid and a well-known immunosuppressive agent. It has been investigated as an effective agent against growth and multiplication of several microbial pathogens including viruses [58]. MPA inhibits the replication of several viruses* in vitro *to varying degrees [59]. MPA is a noncompetitive inhibitor of inosine monophosphate dehydrogenase (IMPDH). MPA is used clinically in the prevention of rejection of transplanted organs [60]. Various studies have reported that MPA blocked the cytopathic effect and replications of several viruses [61].

Khan et al. [62] have assessed the antiviral potential of MPA against CHIKV via inhibition of IMPDH enzyme in vero cells. Inhibition of virus induced apoptosis was observed as measured by caspase-3, PARP, and Bcl-2. Total genome infectivity was determined by analyzing the ratio of total infectious viral particles to the genome copy number. Nontoxic concentration of MPA (10 *μ*M) reduced ≥99.9% CHIKV titre in vero cells. MPA via depletion of substrate for polymerase (GTP) inhibited CHIKV-induced apoptosis.

### 4.4. Viperin

The antiviral role of viperin has been demonstrated in CHIKV infection by studying the longitudinal transcriptional profiles of the innate immune response in PBMCs from a cohort of 24 CHIKV-infected patients. Results showed that type I IFNs controlled CHIKV infection via RSAD2 (which encodes viperin), an enigmatic multifunctional IFN-stimulated gene (ISG). Viperin was highly induced in monocytes, the major target cell of CHIKV in blood.* In vivo* study demonstrated the direct role of viperin in controlling CHIKV replication. Mice lacking Rsad2 had higher viremia and severe joint inflammation compared with wild-type mice [63].

### 4.5. Human Antibodies

Immunotherapy, in the form of human polyclonal antibody, has been used for treatment of human viral infections, and in alphavirus-infected animal models, passive immunization with convalescent sera from animals was protective. Sera from patients and monkeys who have recovered from acute alphavirus-infections have been shown to contain neutralizing antibodies in suckling mouse models. Human polyclonal antibodies (CHIKV Ig) have been purified from plasma of convalescent donors and used in mouse models of CHIKV infection [64]. Results are promising with CHIKV Ig showing both prophylactic and therapeutic potential. In both IFN-*α*/*β*R-/- and immune-competent mouse neonates, a single prophylactic dose of CHIKV Ig was found to be protective against lethality associated with CHIKV, with undetectable viral levels in the serum and no dissemination to the central nervous system. The degree of protection correlated to the dose of antibodies administered. CHIKV Ig also has a therapeutic effect as it is protective against lethality when given up to 8 hours postinfection. The results also supported the hypothesis that viremia precedes CNS dissemination and controlling the viremia prevents neurologic complications [64].

From another point of view, the therapeutic utility of monoclonal/polyclonal antibodies might be limited as patients with a serodiagnosis of an alphaviral disease clearly already have antibodies, and administration of antibodies before endogenous antibody production is likely to be impractical. Nevertheless, such antibodies might find utility in preventing mother-to-child infections. Conceivably, neutralizing antibodies could be used prophylactically during an epidemic. However, parenterally administered antibodies (such as tocilizumab and infliximab) have serum half-lives of only 8–14 days, so repeated dosing would be envisaged in an extended epidemic [65].

A summary of the assays used, the hypothesized target, and the pros and cons of the cellular inhibitors described herein is presented in Table 2.

## 5. High Throughput Screening and Structure Based Drug Discovery Approaches

Recently, cell-based high throughput assays have been developed to identify potential CHIKV inhibitors. One study reported a focus screen of 356 natural compounds and clinically approved drugs using a CHIKV replicon and a concomitant screen with SFV surrogate infection model [66], while another study screened 3,040 small molecules for inhibitors of CHIKV nsP2 using a novel target-based phenotypic assay approach [67]. Cruz et al. [68] used a cell-based high throughput screening assay using resazurin against a kinase inhibitor library combined with the image-based high content assay approach to identify compounds against CHIKV having novel antiviral activity.

In addition, the availability of the crystal structures of several proteins of the CHIKV RNA genome and other related alphaviruses is encouraging the drug discovery process through target structure-based pharmacophore modeling, virtual library screening, and drug docking approaches. When used prior to experimental screening, it can be considered as a powerful computational filter for reducing the size of a chemical library that can be further experimentally tested [69]. Upon the identification of compound hits through such screening, structural models are further used to develop a structure activity relationship to optimize the compound's activity [70]. Rashad and Keller [71] used* in silico* techniques of virtual screening and docking studies for structure based design towards the identification of novel binding sites and inhibitors for the chikungunya virus envelope proteins. Based on multiple enzymatic activities and functional roles of the nsP2 [72, 73], recent studies have considered it as a potential target for CHIKV inhibitors [48, 56]. Bassetto et al. [74] used a bioinformatics approach considering a homology-based model of CHIKV virus based on the crystal structure of nsP2 of the alphavirus VEE as a template. The study identified a few compounds that selectively inhibited CHIKV in a virus-cell based CPE reduction assay, though further experimental studies are ongoing to prove that the compounds are indeed nsP2 inhibitors. Lucas-Hourani et al. [67], on the other hand, used a phenotypic assay to identify one natural compound that partially blocks nsP2 activity and inhibits CHIKV replication* in vitro*. A recent work [75] showed that the nsP4 protein of CHIKV is involved in the mechanism of action of T-705 (favipiravir) that was seen to inhibit CHIKV replication* in vitro* and* in vivo*. Thus it is imperative to target other viral proteins also for structure-based drug design.

## 6. Antiviral Vaccine Approaches

An alternative approach to virus disease control involves the use of vaccines. There is still no effective vaccine to prevent the disease. In 1967, formalin-inactivated CHIKV vaccines were prepared in chick embryo and suckling mouse brain in 1967 and 1972, and in green monkey kidney cell tissue culture [76, 77]. In 1970, CHIKV vaccine was prepared by tween ether extraction [78]. In 1973, Nakao and Hotta [79] indicated that the UV-inactivated virus was superior to the formalinized virus in regard to its immunogenicity in monkeys. In 2000, Edelman et al. [80] published a phase II assay with an attenuated vaccine (strain obtained from an infected patient during the 1962 outbreak in Thailand).

A number of preclinical CHIKV vaccines have been described, including inactivated virus formulations [81–83], live-attenuated virus vaccines [84–86], chimeric virus vaccines [87], DNA vaccines [88, 89], T cell based peptide vaccine [90], a recombinant adenovirus vaccine [91], subunit protein vaccines [92–94], and a virus-like particle (VLP) formulation [95, 96].

The immunogenic potential of recombinant CHIKV envelope proteins has been evaluated in mice. Recombinant protein elicited a strong humoral response and a balanced Th1/Th2 response. Recombinant CHIKV antigens can be proposed as potent subunit vaccine candidates [97].

Live CHIKV vaccine (CHIKV/IRES) that is highly attenuated yet immunogenic in mouse models and is incapable of replicating in mosquito cells has been developed [98]. The development and evaluation of CHIKV vaccine candidates that were attenuated by deleting a large part of the gene encoding nsP3 or the entire gene encoding 6K have been described. This stable and efficient attenuated CHIKV vaccine candidate can be administered either as viral particles or as infectious genomes launched by DNA [99]. Recently two live-attenuated vaccine candidates based on the insertion of a picornavirus internal ribosome entry site (IRES) sequence into the genome of CHIKV have been generated. These CHIKV/IRES vaccine candidates appear to be safe and efficacious, supporting their strong potential as a human vaccine to protect against CHIKV infection and reduce transmission and further spread [100]. The immunogenicity profile and the efficacy of a novel CHIKV vaccine candidate based on the highly attenuated poxvirus vector modified vaccinia virus Ankara (MVA) expressing the CHIKV structural genes C, E3, E2, 6K, and E1 (termed MVA-CHIKV) have been generated and characterized [101]. MVA-CHIKV was found to be an effective vaccine against CHIKV infection, and it induced strong, broad, highly polyfunctional and long-lasting CHIKV-specific CD8^+^ T cell responses, together with neutralizing antibodies against CHIKV. A recent paper [102] has reviewed several vaccine approaches for CHIKV.

## 7. Conclusions

The explosive chikungunya fever epidemic of 2005 in La Réunion and its rapid dissemination to several countries in the Indian Ocean stressed the need for appropriate CHIKV antivirals. In the sections above, we have tried to explore the different perspectives for chikungunya management and treatment and the effectiveness of these regimens. Though advances in molecular and bioinformatics tools have expedited in identifying novel drug and vaccine candidates, the major thrust that is now required is to explore the efficacy of these for CHIKV treatment. As shown in Tables 1 and 2, evaluation studies in* in vivo* systems and further clinical testing of lead compounds are essential for establishing the effectiveness of the potential compounds.

In the management of CHIKV arthralgia, the differential diagnosis of CHIKV, against presence of other conditions such as chronic rheumatic diseases, would be important to treat patients suffering from arthropathy. Among the chemical compounds that have been attempted for CHIKV, some of the well-known broad-spectrum antivirals like chloroquine, ribavirin, and interferon, which have displayed some efficacy in humans, may prove to be promising. Though there is no evidence supporting the clinical efficacy of ribavirin on CHIKV, the combination with interferon could be subjected to clinical trials for the treatment of CHIKV infections. Further, the* in vitro* use of siRNA or plasmid based shRNAs has shown promising results in the cell based infection model of CHIKV. In spite of this, further studies are warranted to rationalize the therapeutic significance of these siRNAs in primates. The data obtained from the studies related to cellular factors could form the basis for future development of novel immune-based control strategies. The chemical structure of the furin inhibiting peptide could be used as a starting point for development of more novel and active peptidomimetics that target the intracellular furin cleavage step. Compounds that have emerged from virtual library screening and structure-based drug discovery would be expected to display lower toxicity and side effects owing to their selectivity to specific viral targets. Some of the issues related to vaccine development are the requirement of further* in vivo* evaluation, the need for clinical trials, and cost and scalability issues. Though the MVA-CHIKV may be considered as a potential vaccine candidate against CHIKV, considering the essential role for B cells and antibodies in controlling CHIKV infection, eliciting strong humoral response through the use of suitable adjuvants would be vital for improvement of the vaccine efficacy.

Nevertheless, fighting CHIKV is not only a matter of drugs and vaccines. Preventive measures should also be applied to avoid mosquito bites responsible for disease transmission. These simple measures include the use of insect repellents, the minimization of skin surface exposed to mosquito bites, the elimination of standing water where mosquitoes can lay eggs, and the installation of window and door screens. Precise measures need to be implemented by the government in the form of surveillance and awareness programs among people and medical practitioners for better management of the disease and for the prevention of new outbreaks.

## Figures and Tables

**Table 1:** Major tested anti-CHIKV chemical compounds.

Products	Assay type	Hypothesized target	Pros	Cons	References

Chloroquine	*In vitro* (vero cells)	Disrupted endosome-mediated CHIKV internalization, possibly through the prevention of endosomal acidification.	*In vitro* study proved that it blocks the production of proinflammatory cytokines and the proliferation of monocytes, macrophages, and lymphocytes.	*In vivo* study required.	Delogu and de Lamballerie, 2011 [50] Khan et al., 2010 [41]

Ribavirin	Human	Can interact with the intracellular viral RNA production.	Faster resolution of joint and soft tissue manifestations.	Involvement of a small number of patients and lack of planning as randomly distributed patients were not compared with a placebo group.	Ravichandran and Manian, 2008 [44]

6-Azauridine	*In vitro* (vero cells)	Inhibition of orotidine monophosphate decarboxylase, an enzyme involved in the de novo biosynthesis of pyrimidine, cytidine, and thymidine.	Showed a significant inhibition of CHIKV at a low concentration.	The antiviral activity has been difficult to replicate *in vivo. *	Briolant et al., 2004 [46]

Arbidol	*In vitro* (vero and primary human fibroblast cells)	Inhibition of virus mediated fusion and blocking of the viral entry into the target cells through inhibition of glycoprotein conformational changes that are essential for the fusion process.	Well-tolerated with minimal side effects.	Not tested in *in vivo* system.	Delogu et al., 2011 [48]

Harringtonine	*In vitro* (BHK21 cells)	Affects CHIKV RNA production inside the infected cell as well as viral protein expression such as the nsP3 and the E2 proteins.	Minimal cytotoxicity.	Not tested in *in vivo* system.	Kaur et al., 2013 [49]

**Table 2:** Some of the major cellular inhibitors against chikungunya virus.

Cellular factors	Assays	Target/effectors	Pros	Cons	References
Furin inhibitors	*In vitro* (myoblast cells).	Intracellular furin-mediated cleavage of viral envelope glycoproteins: the E2E3 or p62 precursor.	Able to induce a stronger inhibition of viral infection.	Not tested in *invivo* system.	Ozden et al., 2008 [54]

2′,5′-Oligoadenylate synthetase (OAS3)	Human epithelial HeLa cell lines.	Affects CHIKV replication through a RNase L-dependent pathway.	Ability of OAS3 to inhibit alphavirus growth may be important for the development of antiviral molecules against CHIKV.	Cannot rule out the possibility that OAS3-mediated inhibition of CHIKV was also due to a block early in virus life cycle, for example, viral entry and uncoating of virus particles.	Bréhin et al., 2009 [56]

Cellular IMPDH enzyme	*In vitro* (vero cells).	Depletion of intracellular guanosine pool.	CHIKV utilizes IMPDH activity for its growth and multiplication which is a potential and effective target to prevent its infection.	It would be useful to explore similar findings by targeting IMPDH in case of other alphaviruses which are more lethal than chikungunya like Sindbis virus, Semliki forest virus, and so forth.	Khan et al., 2011 [62]

Viperin	*In vivo* (monocytes).	Endoplasmic reticulum.	Critical antiviral host protein that controls CHIKV infection and provides a preclinical basis for the design of effective control strategies against CHIKV.	Large gaps in our understanding of the precise mechanisms at play for viperin to exert such a wide variety of roles within the cell.	Teng et al., 2012 [63]
